# An Unprecedented Metal Distribution in Silica Nanoparticles Determined by Single-Particle Inductively Coupled Plasma Mass Spectrometry

**DOI:** 10.3390/nano14070637

**Published:** 2024-04-06

**Authors:** Juan Han, Xu Wu, Julia Xiaojun Zhao, David T. Pierce

**Affiliations:** 1 Department of Chemistry, University of North Dakota, 151 Cornell Street, Stop 9024, Grand Forks, ND 58202, USA; hannah.juan.han@nmt.edu (J.H.); steven.wu01@usd.edu (X.W.); 2 New Mexico Institute of Mining & Technology, 801 Leroy Place, Socorro, NM 87801, USA; 3 Department of Chemistry, University of South Dakota, 414 E. Clark St., Vermillion, SD 57069, USA

**Keywords:** metal-containing nanoparticles, single-particle inductively coupled plasma mass spectrometry, silica nanoparticles, water-in-oil microemulsion

## Abstract

Metal-containing nanoparticles are now common in applications ranging from catalysts to biomarkers. However, little research has focused on per-particle metal content in multicomponent nanoparticles. In this work, we used single-particle inductively coupled plasma mass spectrometry (ICP-MS) to determine the per-particle metal content of silica nanoparticles doped with tris(2,2′-bipyridyl)ruthenium(II). Monodispersed silica nanoparticles with varied Ru doping levels were prepared using a water-in-oil microemulsion method. These nanoparticles were characterized using common bulk-sample methods such as absorbance spectroscopy and conventional ICP-MS, and also with single-particle ICP-MS. The results showed that averaged concentrations of metal dopant measured per-particle by single-particle ICP-MS were consistent with the bulk-sample methods over a wide range of dopant levels. However, the per-particle amount of metal varied greatly and did not adhere to the usual Gaussian distribution encountered with one-component nanoparticles, such as gold or silver. Instead, the amount of metal dopant per silica particle showed an unexpected geometric distribution regardless of the prepared doping levels. The results indicate that an unusual metal dispersal mechanism is taking place during the microemulsion synthesis, and they challenge a common assumption that doped silica nanoparticles have the same metal content as the average measured by bulk-sample methods.

## 1. Introduction

Metal-containing nanoparticles (MNPs) are a type of engineered nanomaterial finding increased use in fields of catalysis [1,2] sensing [3,4] and medicine [5,6,7], yet this greater use of MNPs raises questions about metal content at the nanoparticle level. Typical methods for MNP characterization, such as UV-visible spectroscopy or conventional inductively coupled plasma-mass spectrometry (ICP-MS), only provide bulk metal content that is an average of many particles contributing to the measured signal. The few traditional methods that provide analysis at the particle level, such as scanning electron microscopy coupled with energy dispersion spectroscopy (SEM-EDS) and nanoparticle tracking analysis (NTA), suffer numerous limitations, including drying artifacts and long analysis times (or low numbers of per-particle analyses) in SEM-EDS [8], or poor selectivity and low sensitivity in NTA [9].

Single-particle ICP-MS (spICP-MS) is a mature method that provides reliable particle-by-particle analysis for large numbers of particles (>10,000) in a relatively short period of time (<3 min) [10]. The method combines the high sensitivity and selectivity inherent to ICP-MS with rapidly timed measurements designed to capture a selected isotope signal from individual nanoparticles (NPs). By introducing nanoparticles to the plasma as a low-concentration aqueous solution (<1 × 10^8^ NPs L^−1^), vaporization, atomization and ionization of the individual particles generates an ion cloud that is sampled by the mass spectrometer and detected as a transient signal pulse (<5 ms) for the selected metal isotope. The intensity of the pulse is proportional to the amount of target metal per nanoparticle, whereas the number of detected pulses during the minutes-long data acquisition period provides the particle number concentration.

To date, most spICP-MS determinations involving MNPs have focused on per-particle or aggregate-particle analysis of one-component materials composed of either metallic elements or metal oxides [10,11,12,13,14]. Gold nanoparticles (AuNPs) have received the greatest attention in this regard, with notable examples including per-particle ICP-MS validations for routine measurements of AuNP number and size distribution [15,16,17], and development of guidelines for measuring number concentrations of agglomerated AuNP samples [18]. Our own group has focused on applications of spICP-MS and AuNPs to develop a biomarker assay for thrombin [19] and to determine ionic mercury in environmental samples at levels well below parts-per-trillion [20].

spICP-MS has been applied far less often to multicomponent MNPs. Our searches discovered only a handful of studies focused on bimetallic Au@Ag core-shell samples [21,22,23] and three recent cases that addressed multimetallic Cu-based alloy NPs [24] and bimetallic Au-Ag [25] and Pt-Pd alloy NPs [26]. The present work tackles per-particle metal determination for a very different type of multicomponent MNP—silica nanoparticles synthesized and doped with metal ions under reverse microemulsion conditions. Since their early adoption for bioseparation and bioanalysis [27], doped silica nanoparticles continue to show promise as reagents for theranostics [28], biophotonics [29] and other bioapplications [30]. Although our spICP-MS work with metal-doped silica NPs began as a simple confirmation of bulk-analysis measurements, the unprecedented per-particle metal distributions we discovered provide important insights into the distribution of ionic dopants during reverse microemulsion synthesis and may prove useful for engineering better silica-based catalysts, optical materials, extended network materials, co-crystalline materials, and possibly drug-delivery agents.

## 2. Materials and Methods

A complete list of abbreviations and variables is provided in Supplementary Materials for the convenience of the reader.

### 2.1. Materials

Tetraethyl orthosilicate (TEOS, 99.999%), tris(2,2′-bipyridyl) dicholororuthenium(Ⅱ) chloride hexahydrate ([Ru(bpy)_3_]Cl_2_·6H_2_O, 99.95%), N-[(3-trimethoxysilyl)propyl]-ethylenediamine triacetic acid trisodium salt (40% in water), polyoxyethylene glycol tert-octylphenyl ether (Triton X-100, 2-(C_8_H_17_)C_6_H_4_(OCH_2_CH_3_)_10_OH, BioXtra), ammonia hydroxide (28.0% NH_3_ in water), n-hexanol (≥99%), cyclohexane (99.5%), acetone (≥99.9%), and ethanol (≥99.5%) were obtained from Sigma-Aldrich (St. Louis, MO, USA). Stock OD = 1 suspensions of spherical gold nanoparticles capped with citrate and having diameters of 30 nm (1.80 × 10^14^ NP L^−1^), 60 nm (2.30 × 10^13^ NP L^−1^), and 150 nm (1.47 × 10^12^ NP L^−1^) were purchased from Nanopartz (Loveland, CO, USA). Stock standard solutions of 100,000 μg L^−1^ ionic ruthenium and of 100,000 μg L^−1^ gold in nitric acid were purchased from Inorganic Ventures (Christiansburg, VA, USA) and were each used to make working standards with 2% optima-grade nitric acid (Radnor, PA, USA) for ICP-MS calibrations. Deionized (DI) water (18.2 MΩ·cm) was produced from a Millipore Synergy (Burlington, MA, USA) purification system and used for all nanoparticle synthesis and all analysis in this work. Grade 4.8 liquid argon was used for plasma and nebulizer operation in all ICP-MS measurements. Grade 5 helium was used as an inert gas in kinetic energy discrimination for all conventional ICP-MS measurements.

### 2.2. Instruments

A Hitachi 7500 transmission electron microscope (Tokyo, Japan) was used to record transmission electron microscope (TEM) images of purchased gold nanoparticles (AuNPs) and synthesized tris(bipyridyl)ruthenium(II)-doped silica nanoparticles (Ru-SiO_2_ NPs). A Zetasizer Nano (Malvern Panalytical, Malvern, UK) was used to measure Zeta potential and hydrodynamic diameter of Ru-SiO_2_ NPs. A PerkinElmer Lambda 1050 UV/VIS/NIR spectrometer (Santa Clara, CA, USA) was used to record UV-visible absorbance spectra of Ru-SiO_2_ NPs and free [Ru(bpy)_3_]^2+^ using matched, 1 cm pathlength quartz cells and DI water as a reference. A Thermo Scientific iCAP Qc quadrupole ICP-MS (Waltham, MA, USA) controlled with Qtegra software (version 2.8.2944.202) was used to perform all conventional and single-particle ICP-MS measurements. Grade 5 helium was utilized as an inert gas when the instrument was operated in kinetic energy discrimination (KED) mode. The sampling interface of this instrument included a Teledyne CETAC ASX560 autosampler (Omaha, NE, USA) operating with a carbon fiber sample probe, a multichannel peristaltic pump operating with PVC tubing, a microflow perfluoroalkoxy nebulizer, and a Peltier-cooled quartz cyclonic spray chamber. To evaluate the performance of this instrument, THERMO-4AREV standard solution obtained from Thermo Scientific (Waltham, MA, USA) was checked daily for a maximum ^59^Co, ^115^In, ^238^U and minimum ^140^Ce^16^O/^140^Ce signal. All single particle measurements with ICP-MS were performed in high-sensitivity standard mode. Microsoft Excel and OriginPro Lab (Northampton, MA, USA) were used for data processing and display.

### 2.3. Synthesis of Metal-Doped SiO_2_ NPs

Samples of Ru-SiO_2_ NPs were prepared in triplicate by a water-in-oil microemulsion method similar to one described in the literature [31]. For each sample, 7.50 mL of cyclohexane, 1.77 mL of Triton X-100, and 1.60 mL of n-hexanol were combined and stirred for 20 min to produce a stable oil-phase solution. A stable microemulsion was formed by adding 240 μL of water solution containing 0.0, 13.3, 26.7, 53.4, or 106.8 mM tris(bipyridyl)ruthenium(II) ([Ru(bpy)_3_]^2+^) and stirring for an additional 20 min. After that, 240 μL of TEOS and 100 μL of ammonia hydroxide were added in 20 min intervals, and the hydrolysis reaction was allowed to proceed with stirring for 24 h. To prevent aggregation of nanoparticles in aqueous solution, they were post-coated with carboxyl groups by adding 100 μL of TEOS and 20 μL of N-[(3-trimethoxysilyl)propyl]ethylenediamine triacetic acid into the microemulsion system. After another 24 h, acetone was added to break the microemulsion, and the post-coated Ru-SiO_2_ NPs were isolated by centrifugation at 11,000 rpm for 15 min. One noteworthy observation for the most heavily doped Ru-SiO_2_ NPs (53.4 and 106.8 mM additions of aqueous [Ru(bpy)_3_]^2+^) was an orange color of the [Ru(bpy)_3_]^2+^ chromophore present in the supernatant solution following centrifugation. This indicated the presence of ionic [Ru(bpy)_3_]^2+^ and, therefore, incomplete uptake at the highest doping levels. The particles were re-suspended and washed three times with ethanol and three times with deionized water. The final Ru-SiO_2_ NPs were dried for 24 h at 120 °C before being re-suspended in purified water to yield a stock solution of MNPs with a mass concentration ($C_{NP}$) of 0.4 mg NPs mL^−1^. From this mass concentration, a particle number concentration (*P*, nanoparticles L^−1^) was determined from the accepted silica NP density ($\rho_{SiO2}$, 1.9 g cm^−3^) [32], and an average MNP volume (${\bar{V}}_{p}$, cm^3^) calculated from the average NP radius (${\bar{r}}_{p}$, nm) measured by TEM and assuming a spherical particle shape (${\bar{V}}_{p}=\frac{4}{3}\cdot \pi {{\cdot \bar{r}}_{p}}^{3}\cdot 10^{-21}$).

(1) $$P=\frac{C_{NP}}{{\bar{V}}_{p}\cdot \rho_{SiO2}}$$

### 2.4. Characterization of MNP Shape and Size

Samples of both Ru-SiO_2_ NPs and AuNPs were prepared for TEM evaluation according to instructions of the UK National Physical Laboratory [33]. Briefly, stock solutions of Ru-SiO_2_ NPs with varied dopant (0.4 mg L^−1^) or purchased 150 nm AuNP standards (1.47 × 10^12^ NP L^−1^) were diluted with DI water to a particle concentration of ca. 10^9^ NPs L^−1^. The diluted solutions were sonicated for 2 min in an ice water bath. Then, a 3.0 μL solution aliquot was dropped onto a copper TEM grid using a micropipette, and the samples were dried at room temperature for 24 h before characterization. Particle shape was consistently and sufficiently spherical for all MNPs that a single diameter measurement ($d_{p}$, nm) was used to characterize each individual particle. The diameter of 200 individual MNPs was measured from TEM images using ImageJ software (version 1.52a) and averaged to determine an average NP radius (${\bar{r}}_{p}$, nm) for each sample.

### 2.5. Determinations of Metal Atoms per Particle

#### 2.5.1. UV-Visible Absorbance Spectrophotometry

This method constitutes a bulk analysis method because the Ru-based absorbance signal is measured for a relatively large volume of solution (ca. 250 μL; 1 cm pathlength × 0.25 cm^2^ illumination area) containing a very large number of individual nanoparticles (minimum number ca. 2 × 10^9^). Solutions of Ru-SiO_2_ NPs and free [Ru(bpy)_3_]^2+^ showed the same strong absorbance centered at 454 nm for the [Ru(bpy)_3_]^2+^ chromophore. Absorbance calibrations were performed at this wavelength for Ru-SiO_2_ NP samples with different Ru doping levels at particle concentrations (P) ranging from ca. 8 × 10^12^ to 2 × 10^14^ NPs L^−1^ and for free [Ru(bpy)_3_]^2+^ ions in solution at concentrations ranging from 1.0 μM to 40.0 μM. All calibration plots showed linear correlations over these concentration ranges (*R*^2^ ≥ 0.99). Moreover, because absorbance spectra (e.g., wavelength maxima, etc.) were very similar for Ru-SiO_2_ NP samples and free [Ru(bpy)_3_]^2+^, it was possible to calculate an average number of Ru atoms per Ru-SiO_2_ NP (${\bar{N}}_{p}$) for each Ru-doping level. Assuming the [Ru(bpy)_3_]^2+^ chromophore has the same absorptivity coefficient free in solution as it does within the SiO_2_ NP [34], a ratio of Ru-SiO_2_ NP calibration slope ($m_{p}=A_{454}\cdot P^{-1}$) to the free [Ru(bpy)_3_]^2+^ calibration slope ($m_{free}=A_{454}\cdot {\mu M}^{-1}$) yields ${\bar{N}}_{p}$ when multiplied by Avogadro’s number ($N_{A}$), as shown in Equation (2).

(2) $${\bar{N}}_{p}=\frac{m_{p}}{m_{free}}\cdot N_{A}\cdot 10^{-6}$$

#### 2.5.2. Conventional ICP-MS

Conventional operating conditions for ICP-MS are listed in Table 1. Analysis was performed in KED mode for all experiments, and all other instrument parameters were optimized to meet requirements defined by the manufacturer prior to method calibration and analysis. Ru-SiO_2_ NP samples with different Ru doping levels were prepared at a particle concentration (P) of ca. 4 × 10^9^ NPs L^−1^ in 2% nitric acid and were combined with 10 μg L^−1^ of Ge and Bi internal standards in 2% nitric acid from separate pump channels and introduced together into the ICP-MS nebulizer. The integrated ^102^Ru signal (counts s^−1^) was monitored relative to the internal standards (^74^Ge and ^209^Bi) and calibrated using ionic Ru standards ranging from 0.05 to 5.0 μg L^−1^ in 2% nitric acid.

As with absorbance spectrophotometry, this conventional method of ICP-MS analysis (with its long 50 ms dwell/integration time and 10 averaged sweeps) constitutes a bulk analysis method because the ^102^Ru signal is integrated and averaged for a sizable solution volume (ca. 2 μL, 0.2 mL min^−1^ sample flow rate × 0.05 s dwell time × 10 sweeps) containing a large number of individual nanoparticles (minimum number ca. 8 × 10^3^). The integrated and averaged ^102^Ru signal yielded an average mass concentration of Ru in solution ($C_{Ru}$, μg L^−1^) when calibrated to the ionic Ru standards, and an average number of Ru atoms per Ru-SiO_2_ NP (${\bar{N}}_{p}$) was determined as shown in Equation (3), where $M_{Ru}$ is the molar mass of Ru.

(3) $${\bar{N}}_{p}=\frac{C_{Ru}}{P}\cdot \frac{N_{A}}{M_{Ru}}\cdot 10^{-6}$$

Accuracy of these bulk MNP measurements was confirmed by complete digestion of triplicate Ru-SiO_2_ NP samples spanning the entire metal-doping range and analysis of the digests using the same conventional ICP-MS conditions used for MNP solutions. See Supplementary Materials for digestion procedure and analysis results (Table S1).

#### 2.5.3. Single-Particle ICP-MS

Instrument operating conditions are listed and compared to conventional ICP-MS conditions in Table 1.

Conversion to a high-sensitivity standard acquisition mode (STDS) was required for single-particle measurements, as was physical replacement of the cone separator insert and sample probe. Time-resolved data acquisition was controlled using a Qtegra software plug-in. Calibration and quality-control steps typically followed the RIKILT Standard Operating Procedure for counting and sizing of nanoparticles [35]. Sample flow rate (u, ca. 0.20 mL min^−1^) was measured daily in triplicate by weighing 600 s of water uptake. The method of Pace et al. [36] was followed closely to measure both nebulizer transport efficiency ($\eta_{n}$, 8.9%, with ±0.1% variation for 30, 60 and 150 nm AuNPs) and MNP ionization efficiency ($\eta_{i}$, 65% for 150 nm AuNPs and 100% for Ru-SiO_2_ NPs). See Supplementary Materials for exact procedures and associated results (Table S2). Samples were diluted to a low concentration of 5.0 × 10^7^ NPs L^−1^ with high-purity water, and the chosen isotope (^197^Au for AuNP and ^102^Ru for Ru-SiO_2_ NP) was measured in units of counts per dwell time. A short 5 ms dwell/integration time ($t_{d}$, ms) and sampling interval (τ) of 180 s (corresponding to 36,000 individual measurements) were used in most experiments. The combined conditions of low particle concentration and short dwell/integration time allowed discrete per-particle analysis of metal content.

Because less than 10% of the 36,000 individual dwell-time measurements during a 180 s sampling interval corresponded to individual MNPs, a detection threshold using a five times standard deviation criterion (Equation (4)) was used to differentiate measurements of Ru-SiO_2_ NPs ($I_{p}$, counts per dwell time) from background noise. The usual iterative algorithm that averages the nanoparticle data set and selects $I_{p}$ signals above the threshold [36,37] did not work in our case. According to Tuoriniemi et al. [38], the iterative algorithm converges to a proper detection threshold for NPs (effectively, Equation (4)) with Gaussian or Poisson signal distributions—the usual case with single-component MNPs. However, our Ru-SiO_2_ NPs demonstrated a geometric signal distribution (similar to a noisy background), so the iterative algorithm missed many low $I_{p}$ signals that significantly exceeded actual blank solution measurements. As such, we applied a threshold criterion (Equation (4)) that used averaged measurements of a blank data set (${\bar{I}}_{blank}$, counts per dwell time) plus five times the blank signal standard deviation ($\sigma_{blank}$, counts per dwell time) [39].

(4) $$I_{p}>{\bar{I}}_{blank}+5\cdot \sigma_{blank}$$

Measurements of each detected MNP in counts per dwell time ($I_{p}$) were converted to number of metal atoms per nanoparticle ($N_{p}$) by a calibration procedure like the one of Pace et al. [36]. Briefly, average counts per dwell time (${\bar{I}}_{x}$) were measured for a series of ionic calibration standards with mass concentration $C_{x}$ (0.1–10.0 µg L^−1^ for *x* = Au and 0.05–5.0 µg L^−1^ for *x* = Ru) and molar mass $M_{x}$, and then plotted versus the number of metal ions entering the plasma per dwell time ($N_{x}$, Equation (5)).

(5) $$N_{x}=C_{x}\cdot \frac{N_{A}}{M_{x}}\cdot u\cdot \eta_{n}\cdot t_{d}\cdot 10^{-6}$$

Slope *m* of this linear plot (forced y-intercept *b* = 0) yielded an expression for the number of metal atoms per nanoparticle, Equation (6).

(6) $$N_{p}=\frac{I_{p}}{m\cdot \eta_{i}}$$

Finally, to directly compare per-particle measurements of $N_{p}$ (spICP-MS) with average measurements (${\bar{N}}_{p}$) obtained from bulk measurements (UV-Vis and conventional ICP-MS), the *n* measurements of $N_{p}$ over one sampling interval were averaged as shown in Equation (7).

(7) $${\bar{N}}_{p}=\frac{\sum_{i=1}^{n}N_{p}}{n}$$

For some samples, spICP-MS was also used to measure the number (*n*) of detected MNPs per sampling interval (τ) to determine a particle number concentration (P, NPs L^−1^) from Equation (8).

(8) $$P=\frac{n}{u\cdot \eta_{n}\cdot \tau }\cdot 10^{3}$$

### 2.6. Histogram Distributions

Single-particle ICP-MS. For display, $I_{p}$ measurements of detected MNPs (using the threshold defined in Equation (4)) were processed using OriginPro Lab as a histogram with number of MNPs (plotted on y-axis) falling within a discreet interval of $I_{p}$ value (plotted on x-axis). A histogram x-axis interval (also called the histogram bin size) of 5 counts per dwell time was used in most cases. $I_{p}$ data presented in this manner were processed further to display the distribution in number of metal atoms per detected MNP ($N_{p}$ using Equation (6)).

## 3. Results

### 3.1. Synthesis and Characterization of Ru-SiO_2_ NPs

Metal-doped silica nanoparticles are typically prepared by either the water-in-oil microemulsion method or the Stöber method [40,41,42]. The former produces smaller MNPs with a more narrow size distribution, so it was used to synthesize the Ru-SiO_2_ NP samples in this work. Another benefit of this method is the ease by which the metal amount can be varied by changing the dopant concentration in the water used to make the microemulsion. For this work, [Ru(bpy)_3_]^2+^ concentrations were varied from 0.0 mM (control, dopant level 0) to 13.3 mM (dopant level 1), 26.7 mM (dopant level 2), 53.4 mM (dopant level 3), and 106.8 mM (dopant level 4). The nascent samples were also post-coated with COOH groups to inhibit aggregation in aqueous suspension.

**Figure 1:**
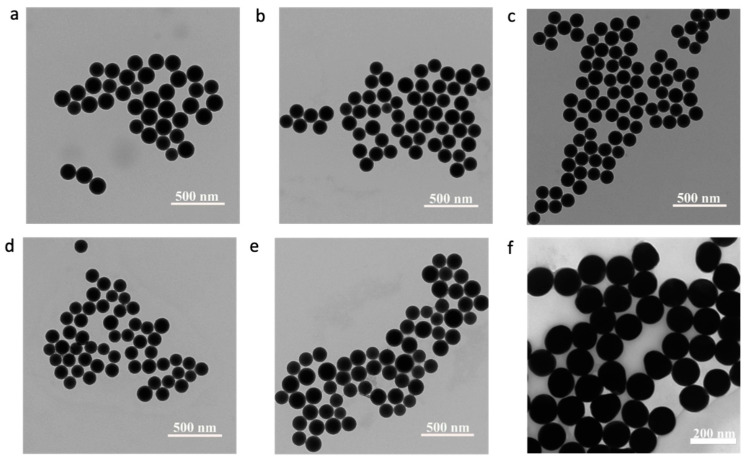
TEM images of Ru-SiO_2_ NPs with different doping levels (**a**–**f**) and commercial 150 nm AuNPs (**f**). [Ru(bpy)_3_]^2+^ concentrations used to prepare the different Ru-SiO_2_ NP samples were 0.0 mM ((**a**), control, dopant level 0), 13.3 mM ((**b**), dopant level 1), 26.7 mM ((**c**), dopant level 2), 53.4 mM ((**d**), dopant level 3), and 106.8 mM ((**e**), dopant level 4).

TEM images of these samples (Figure 1a–e) showed the NPs were spherical with average diameters of 148 ± 9 nm (control), 139 ± 8 nm (1), 128 ± 7 nm (2), 127 ± 8 nm (3), and 151 ± 16 nm (4).

**Figure 2:**
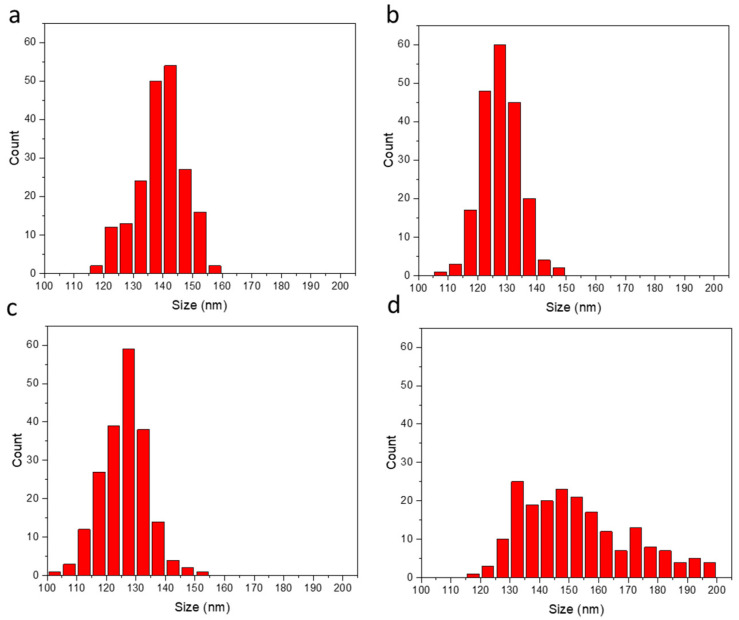
Size distributions derived from TEM images of Ru-SiO_2_ NPs with different doping levels (**a**–**d**). Diameters for 200 different NPs of each sample were measured and counted for each 5 nm size interval. [Ru(bpy)_3_]^2+^ concentrations used to prepare the different Ru-SiO_2_ NP samples: 13.3 mM ((**a**), dopant level 1), 26.7 mM ((**b**), dopant level 2), 53.4 mM ((**c**), dopant level 3), and 106.8 mM ((**d**), dopant level 4). Bin size 5 nm.

Except for the highest dopant level (4), the NP samples demonstrated a normal distribution (Figure 2a–d) and a size variation that was comparable to commercial AuNPs of roughly the same diameter (Figure 1f, 126 ± 8 nm). The significantly broadened distribution for the highest dopant level (Figure 2d) indicates some disruption of the reverse microemulsion used for synthesizing these silica NPs.

**Figure 3:**
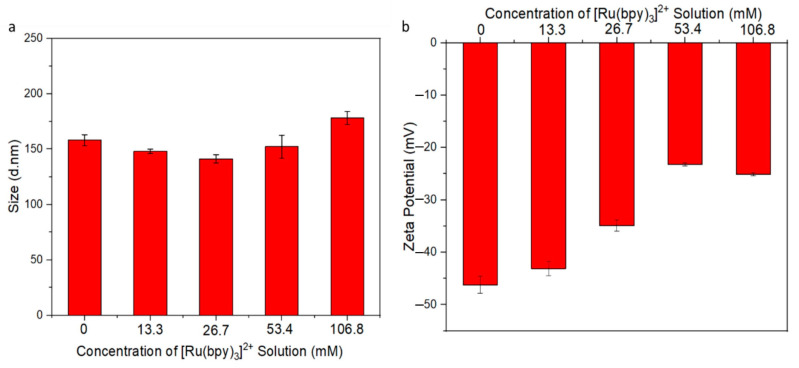
Dynamic light scattering (**a**) and Zeta potential (**b**) measurements of Ru-SiO_2_ NPs with different doping levels. Columns with error bars represent the average with standard deviation for measurements of three separate samples. [Ru(bpy)_3_]^2+^ concentrations used to prepare the different Ru-SiO_2_ NP samples were 0.0 mM (control, dopant level 0) 13.3 mM (dopant level 1), 26.7 mM (dopant level 2), 53.4 mM (dopant level 3), and 106.8 mM (dopant level 4).

Size and monodispersion of the prepared Ru-SiO_2_ NPs in aqueous solution were also evaluated by dynamic light scattering (DLS) and Zeta potential measurements. Average hydrodynamic particle diameters of 158 ± 5 nm (control, dopant level 0), 148 ± 2 nm (dopant level 1), 141 ± 4 nm (dopant level 2), 152 ± 10 nm (dopant level 3), and 178 ± 6 nm (dopant level 4) in Figure 3a were in adequate agreement with TEM measurements, and size distributions showed no indication of particle aggregation. Zeta potentials (Figure 3b) were consistently less than −20 mV, which indicated a robust carboxylate post-coating for all samples that ensured good dispersal of the NPs in solution.

### 3.2. Average Metal Content of Ru-SiO_2_ NPs

**Figure 4:**
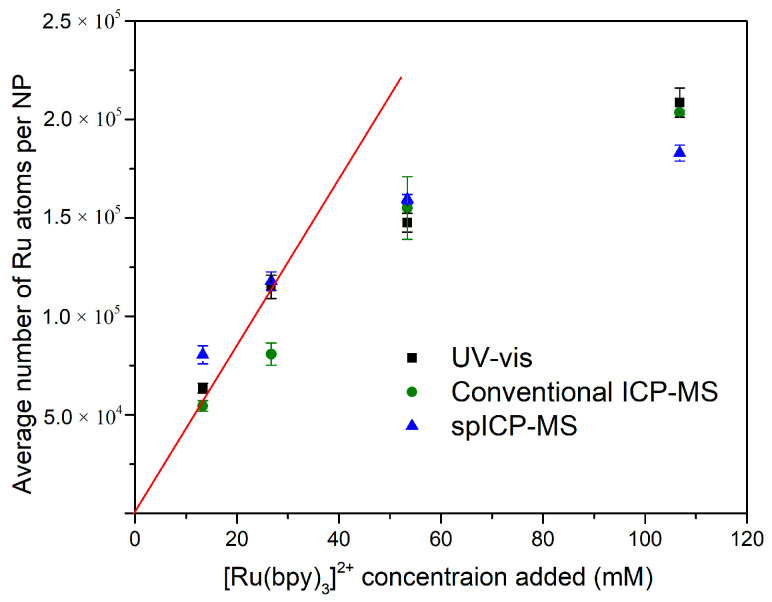
Relationship between the average number of Ru atoms measured per NP (${\bar{N}}_{p}$) for Ru-SiO_2_ NPs synthesized with different doping levels. Measurements of ${\bar{N}}_{p}$ include bulk methods: UV-vis (Equation (2), rectangle) and conventional ICP-MS (Equation (3), circle); and spICP-MS ((Equation (7), triangle). [Ru(bpy)_3_]^2+^ concentrations used to prepare the different Ru-SiO_2_ NP samples were 13.3 mM (dopant level 1), 26.7 mM (dopant level 2), 53.4 mM (dopant level 3), and 106.8 mM (dopant level 4). The line is a fit of the first two doping levels with a forced y-intercept of zero.

All three analysis methods used in this study provided an average number of Ru atoms per NP (${\bar{N}}_{p}$) because each method includes or can combine many particles (≥2500) in the measurement. For the bulk sample methods (UV-vis and conventional ICP-MS), the volume of NP solution analyzed for one measurement is relatively large, so large numbers of NPs are already included. For spICP-MS, the volume for one measurement is much smaller, but many individual particle measurements can be averaged over a sampling interval (Equation (7)). Figure 4 compares ${\bar{N}}_{p}$ results for all three methods, and it is notable how closely the results correspond despite the very different measurement conditions and different metal doping levels. Also evident is a roughly linear increase in ${\bar{N}}_{p}$ when low concentrations of [Ru(bpy)_3_]^2+^ were used in the microemulsion synthesis (red line). However, ${\bar{N}}_{p}$ values did not increase nearly as much for the highest [Ru(bpy)_3_]^2+^ concentrations, suggesting dopant uptake is especially poor at those levels. This conclusion is also supported by observations that free [Ru(bpy)_3_]^2+^ was recovered during synthesis of the most highly doped Ru-SiO_2_ NPs.

### 3.3. Per-Particle Metal Content of Ru-SiO_2_ NPs

Because multicomponent MNPs have a much lower metal content per particle than single-component MNPs of the same size, they present a challenge for per-particle analysis. Measurement dwell/integration time ($t_{d}$, ms) must be chosen carefully [43,44]. Short dwell times improve signal-to-noise for MNP quantification, but they also increase the likelihood of splitting a single-particle signal between two adjacent integration periods [36]. This signal splitting can cause negative bias in measurements of the average number of metal atoms per NP (${\bar{N}}_{p}$, Equation (7)), over-counts of detected MNPs, and positive bias in measured particle number concentration (P, Equation (8)). The Ru-SiO_2_ NPs studied in this work required the shortest possible dwell time of 5 ms to achieve a sufficient ^102^Ru signal-to-noise ratio for quantification. However, a negative bias in average number of Ru atoms per NP (${\bar{N}}_{p}$) was not evident from comparisons with bulk sample methods (Figure 4), except possibly for samples with the highest Ru doping (level 4). These samples had particles with significantly larger diameter (Figure 2d)—a factor that makes MNPs more prone to signal splitting in spICP-MS [45].

**Figure 5:**
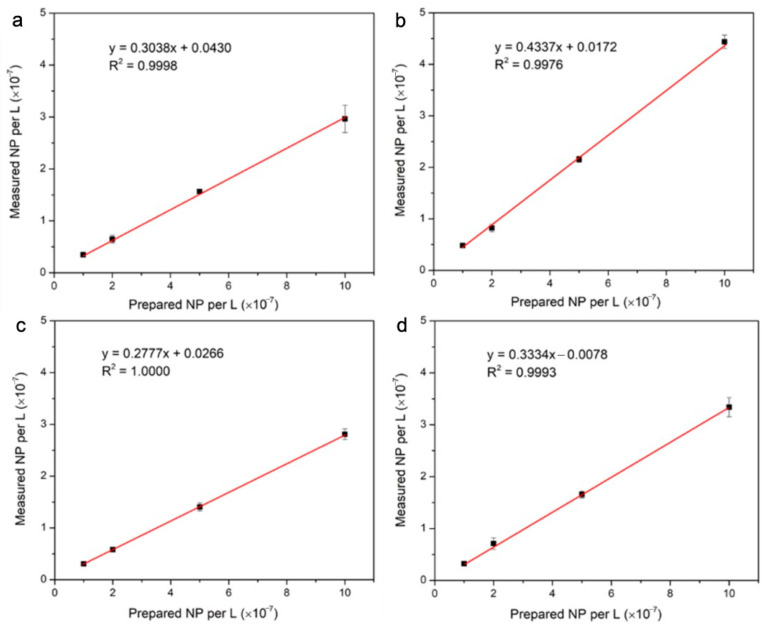
Correlations between particle number concentrations (P, NPs L^−1^) measured by spICP-MS and prepared from four different Ru-SiO_2_ NP samples with [Ru(bpy)_3_]^2+^ dopant level 1 (a), dopant level 2 (b), dopant level 3 (c), and dopant level 4 (d). Each measured particle number concentration is the average (with ±standard deviation as error bars) for three different Ru-SiO_2_ NP solutions prepared with the same [Ru(bpy)_3_]^2+^ dopant level. spICP-MS conditions: measured isotope ^102^Ru, dwell time 5 ms, sampling period 180 s.

Evidence of signal splitting was further evaluated by comparing measured particle number concentrations (Equation (8)) with prepared particle number concentrations (Equation (1)). Correlations for the different Ru-SiO_2_ NP samples (Figure 5) were consistently linear, indicating good particle stability and monodispersivity over the particle concentrations tested. However, slopes (*m*) for all doping levels were significantly less than one (*m* = 0.25–0.43). This result is contrary to a signal splitting effect, which would yield particle number slopes greater than one. The most likely interpretation is based on the geometric metal distributions discovered for these doped silica NPs (vide infra). Such a distribution results in more than half of the Ru-SiO_2_ NPs in each sample having a metal content below the detection threshold of our spICP-MS instrument and so are not counted in the measured particle concentrations.

**Figure 6:**
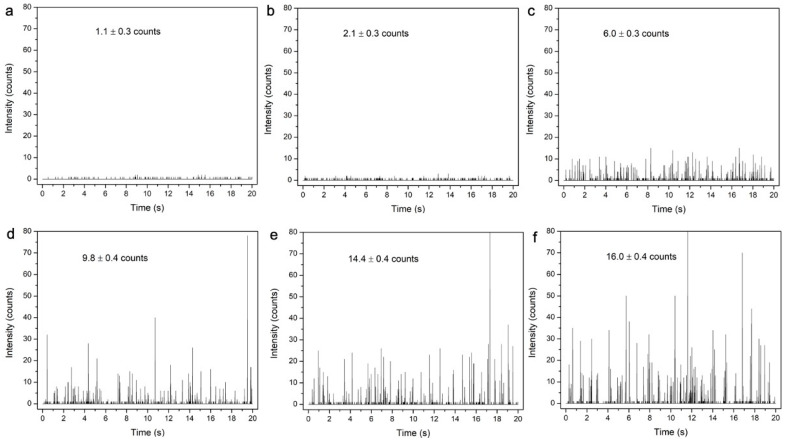
Time-base plot of raw counts measured by spICP-MS for Ru-SiO_2_ NPs prepared with different metal doping levels. Blank solution without Ru-SiO_2_ NPs ((**a**), control), and Ru-SiO_2_ NPs with dopant level 0 ((**b**), control), dopant level 1 (**c**), dopant level 2 (**d**), dopant level 3 (**e**), and dopant level 4 (**f**). Conditions: measured isotope ^102^Ru, dwell time 5 ms, sampling period 180 s, 5.0 × 10^7^ NPs L^−1^ for each sample. Inset values are counts averaged over the entire sampling period for three replicate sample solutions (±standard deviation, N = 3 × 36,000 = 108,000).

**Figure 7:**
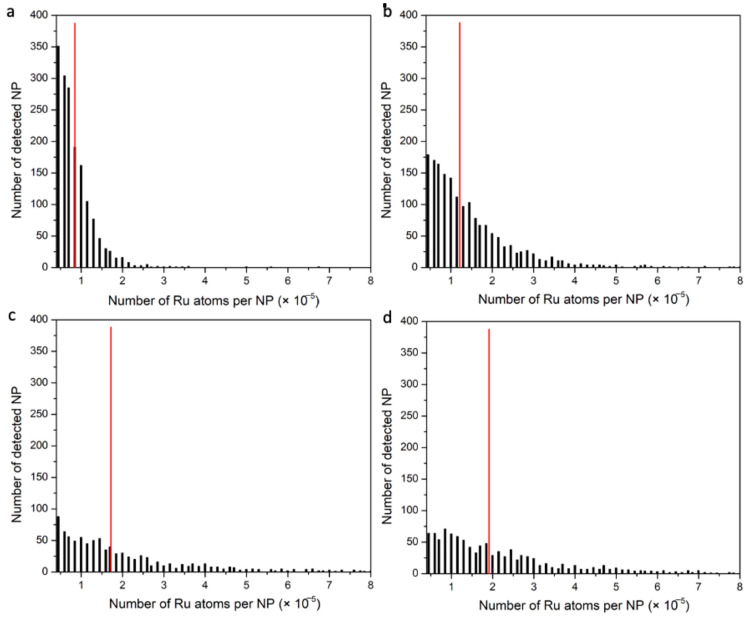
Distribution histograms of $N_{p}$ values (Ru atoms per NP, bin size 5000) measured by spICP-MS for Ru-SiO_2_ NPs prepared with different metal doping levels. (**a**) dopant level 1, (**b**) dopant level 2, (**c**) dopant level 3, and (**d**) dopant level 4. Red lines indicate the average number of Ru atoms per NP (${\bar{N}}_{p}$) determined by conventional ICP-MS for the same sample. Conditions: measured isotope ^102^Ru, dwell time 5 ms, sampling period 180 s, 5.0 × 10^7^ NPs L^−1^ for each sample.

Figure 6 shows partial time-base plots of ^102^Ru counts measured by spICP-MS for Ru-SiO_2_ NPs with increasing metal-dopant levels. Baselines for the Ru-SiO_2_ NP samples (Figure 6c–f) are the same as the blank and control samples (Figure 6a,b), confirming that leakage of free [Ru(bpy)_3_]^2+^ from the doped NPs did not influence the spICP-MS measurements. Signal intensities for Ru-SiO_2_ NPs are readily detectable above the baseline noise, and the time-based plots are like ones obtained for a single-component MNP like AuNPs. However, when individual values of Ru atoms per particle ($N_{p}$) are combined in histogram distributions for each dopant level (Figure 7a–d), the plots are quite different from single-component MNPs. As a crucial reference point, the *average* number of Ru atoms determined per NP (${\bar{N}}_{p}$) by conventional ICP was also plotted as a red line on the corresponding histograms.

The most striking feature of these plots is the lack of *any* histogram peak for *any* doping level. Because the size of the Ru-SiO_2_ NPs is quite homogeneous (Figure 2), a peak in the histogram distribution should appear very close to the ${\bar{N}}_{p}$ value if the concentration of [Ru(bpy)_3_]^2+^ per NP was the same from particle to particle. Instead, the histogram distributions for Ru-SiO_2_ NPs show the highest number of detected NPs with the smallest number of Ru atoms, and exponentially smaller numbers of NPs with much higher numbers of Ru atoms. This type of histogram pattern is consistent with a geometric distribution of metal content per particle, or possibly a lognormal distribution with the zero-leg falling below the particle detection limit (Equation (4)). It is important to note that neither distribution has been reported previously, even for other multicomponent MNPs [22,23,24,25,26].

**Figure 8:**
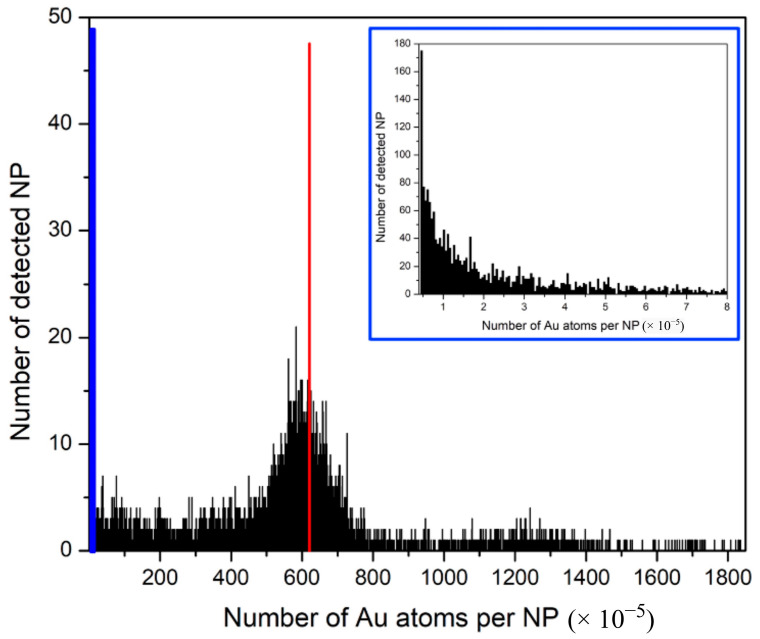
Distribution histograms of $N_{p}$ values (Au atoms per NP, bin size 5000) measured by spICP-MS for commercial AuNPs. Red line is the calculated ${\bar{N}}_{p}$ = 620 × 10^5^ Au atoms per NP determined by the AuNP density and average diameter of 126 ± 8 nm. The lowest $N_{p}$ region (boxed in blue) is shown expanded with inset. Conditions: measured isotope ^197^Au, dwell time 5 ms, sampling period 180 s, 5.0 × 10^7^ NPs L^−1^.

To emphasize the unusual histogram distributions measured for Ru-SiO_2_ NPs, a histogram distribution measured for commercial AuNPs with roughly the same size as the Ru-SiO_2_ NPs and using the same spICP-MS conditions is shown in Figure 8. Although many split-particle measurements with low $N_{p}$ values are evident in this histogram due to the short 5 ms dwell time used (boxed region of Figure 8 shown as inset in blue), a Gaussian peak is clearly observed. Moreover, this peak is centered very close to the average Au atoms per NP (${\bar{N}}_{p}$ = 620 × 10^5^, red line in figure) determined from bulk Au density ($\rho_{Au}$, 19.3 g cm^−3^) and the average particle diameter measured by TEM (Figure 1f, 126 ± 8 nm) and the statistically equivalent ${\bar{N}}_{p}$ value (603 × 10^5^) determined by conventional ICP-MS. This type of Gaussian distribution is invariably measured for single-component MNPs with consistent size [10,45] because metal concentration per particle is simply governed by the physical density (mass per unit volume) of the metallic element or compound, and density is constant from particle to particle.

Another noteworthy characteristic of the unusual histograms in Figure 7 is that a higher [Ru(bpy)_3_]^2+^ doping level increases the number of NPs with more Ru atoms and decreases the number NPs with fewer Ru atoms. This effect broadens and flattens the distribution, resulting in a higher average number of Ru atoms per NP (e.g., Figure 4).

## 4. Discussion

The unusual geometric distributions of $N_{p}$ measured for Ru-SiO_2_ NPs (Figure 7) are clearly not the result of geometric particle size variation (e.g., TEM results in Figure 1 and Figure 2). They are also unlikely to be the result of instrument or measurement artifacts. The close correspondence between *average* metal content per NP (${\bar{N}}_{p}$) determined by spICP-MS and bulk methods for a wide range of dopant levels (Figure 4) could not occur unless a sufficiently accurate set of per-particle determinations was averaged for a sufficiently representative and large number of particles. A possible criticism of the instrument used for these spICP-MS measurements (Thermo iCAP Qc, *circa* 2015) is its relatively slow signal acquisition by today’s standards, which limits dwell times (the ion-count integration period) to the millisecond timescale. Because this integration period is longer than the NP transit time in the plasma, great care must be taken to minimize signal bias due to simultaneous measurement of two or more NP signals within a single dwell time, or only partial measurement of an NP signal if it occurs at the beginning or end of the dwell period. Such care was taken in this work by always using low particle concentrations (<1 × 10^8^ NPs L^−1^) and critically evaluating spICP-MS results using control methods (Figure 4), control measurements (Figure 5), and control samples (Figure 8). It should also be noted that early spICP-MS work with MNPs (2000s [46] through middle 2010s [47]) was performed exclusively using instruments with millisecond acquisition times. Those results yielded both accurate and ground-breaking per-particle quantification. While a faster instrument with microsecond acquisition times would provide better sampling of each particle event and higher accuracy for histogram values close to the $N_{p}$ detection limit (Equations (4) and (6)) [48], a millisecond instrument is certainly adequate to identify unusual distributions such as those exhibited in Figure 7, if only at a semiquantitative level [16].

**Figure 9:**
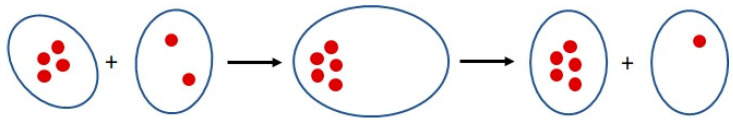
Schematic diagram picturing cooperative exchange of dopant molecules (red spheres) between two fusing/splitting micelles of a dynamic microemulsion.

The remaining and most likely cause for these unusual Ru-doping variations at the particle level appears to be uneven distribution of metal dopant during the microemulsion synthesis. Up to now, synthesis of metal-doped SiO_2_ NPs using water-in-oil microemulsions has been assumed to produce NPs with a consistent metal-doping concentration per particle. This is because the water solution used to form the microemulsion has a homogenous concentration of metal dopant, and it is assumed this homogenous concentration is carried through to the nanodroplet micelles that comprise microemulsion. However, a few computational studies have suggested that rapid inter-micellar exchange of dopant in dynamic nanodroplet micelles can lead to inhomogeneous distributions if the dopant undergoes precipitation [49,50]. Factors that favor the formation of inhomogeneous distributions of dopant within micelles are kinetic in nature—specifically, when inter-micellar exchange of dissolved dopant is fast and precipitate nucleation is slow compared to growth. Under these conditions, the few micelles that manage to nucleate a dopant precipitate tend to accumulate even more from the many adjacent micelles that have yet to form a precipitate. Jain and Mehra modeled this process as a cooperative exchange [47], whereby dopant within two fused micelles preferentially moves into the micelle that originally contained the higher dopant level due to precipitation (Figure 9).

This mechanism leads to most micelles having less dopant than the initial concentration and a much smaller number that accumulate a large amount of dopant precipitate. Because the overall distribution predicted for this mechanism is geometric—the same dopant distribution shown in Figure 7 for Ru measured by spICP-MS—it seems likely this mechanism is active in the microemulsion synthesis of Ru-SiO_2_ NPs. This conclusion is supported by additional observations that (i) relatively high concentrations of [Ru(bpy)_3_]^2+^ dopant solution were used to make these NPs (10–100 mM), (ii) the [Ru(bpy)_3_]Cl_2_ dopant has a limited solubility in water solutions to begin with (ca. 1% by weight), and (iii) solubility of the ionic dopant is probably even lower within the nanodroplet micelles because of the water-in-oil environment.

## 5. Conclusions

This work has demonstrated the first use of spICP-MS to measure per-particle metal composition of a common class of multicomponent MNP—metal-doped silica nanoparticles prepared by the water-in-oil micro-emulsion method. This type of MNP has been used for years as a fluorescence-labeling reagent in many applications and up to now has been considered a very well-understood nanomaterial. However, the most important finding of this work is the highly unusual per-particle distribution of metal content in these Ru-SiO_2_ NPs, which up to now has been assumed to be homogeneous across all particles. One reason why these and other MNPs are often assumed to have homogeneous metal content is a built-in bias of the bulk-analysis methods typically used for their characterization—methods such as UV-visible absorbance or fluorescent spectroscopy or conventional ICP-MS. These bulk methods, by their nature, can only determine an average metal content for the many individual MNPs that produce the measurement signal. Only a particle-by-particle analysis method such as spICP-MS can identify inhomogeneity in metal content, and this work clearly demonstrates why such as method should be added to the routine screening of any new MNP—especially one with more than one component. It seems remarkable that per-particle testing of chemical composition has been largely ignored for routine characterization of MNPs, while per-particle testing of morphology via SEM and/or TEM has long been considered mandatory. However, this will likely change with fast and sensitive analytical methods, such as spICP-MS, now available for routine nanomaterial screening.

A related insight from this work is that correspondence between average metal content per NP (${\bar{N}}_{p}$) determined by spICP-MS and bulk methods (e.g., Figure 4) must be checked, and they must be the same, or at least very similar. Such correspondence indicates that the spICP-MS method provides sufficiently accurate per-particle results that, when averaged for a sufficiently large number of particles, match closely with the inherent averages determined by the bulk-analysis methods. Accordingly, bulk-analysis methods should continue to be part of any routine characterization of MNPs because their results provide useful information about sample-to-sample reproducibility. However, they also play an indispensable role in validating per-particle results provided by spICP-MS.

Finally, our work demonstrates that spICP-MS analysis can provide important mechanistic insights into nanoparticle synthesis in general and into microemulsion behavior specifically. In the future, spICP-MS measurements could be used to develop novel micellar strategies for controlling supramolecular assembly (e.g., metal-organic frameworks) or possibly co-crystallization.

## Figures and Tables

**Table 1:** ICP-MS operating conditions used for conventional and single-particle measurements.

Parameter	Conventional	Single-Particle
Sample introduction		
peristaltic pump	4-channel, 12-roller	4-channel, 12-roller
pump speed (rpm)	20	20
sample tubing (mm ID)	0.508	0.508
internal-standard tubing (mm ID)	0.508	not used
waste tubing (mm ID)	1.295	1.295
nebulizer	Microflow PFA-ST	Microflow PFA-ST
nebulizer gas flow (L/min)	1.09	1.05
spray chamber	quartz cyclonic	quartz cyclonic
spray chamber temperature (°C)	2.70	2.70
Plasma		
torch	ICAP Q quartz	ICAP Q quartz
Rf power (W)	1550	1550
coolant gas flow (L/min)	14	14
plasma gas flow (L/min)	8	8
sample injector	quartz (2.5 mm ID)	quartz (2.5 mm ID)
Mass spectrometer		
sample cone	nickel	nickel
skimmer cone	nickel	nickel
cone insert (mm)	3.5	2.8
mode	KED	STDS
KED gas flow (mL/min)	4.6	0
dwell time (ms)	50	5
averaged sweeps	10	0
internal standards	^74^Ge, ^209^Bi	none

## Data Availability

The raw data supporting the conclusions of this article will be made available by the authors on request.

## Supplementary Materials

The following supporting information can be downloaded at: https://www.mdpi.com/article/10.3390/nano14070637/s1, S1. Abbreviations and variables used in this manuscript; S2. Digestion and conventional ICP-MS analysis of Ru-SiO_2_ NPs; Table S1. Ru determination by conventional ICP-MS for solutions of Ru-SiO_2_ NPs with different Ru-doping levels and pretreatments; S3. Transport efficiency method and results for Au NPs of various sizes; S4. Ionization efficiency method and results for MNPs; Table S2. Percent ionization efficiency ($\eta_{i}$) for each Au NP diameter by conventional ICP-MS using 1 μg NPs L^−1^ solutions of digested and undigested Au NPs. Refs. [51,52,53] are cited in the Supplementary Materials.
