# Supplementary material for: An Unprecedented Metal Distribution in Silica Nanoparticles Determined by Single-Particle Inductively Coupled Plasma Mass Spectrometry
Source: Nanomaterials (Basel). 2024 Apr 6;14(7):637. doi: 10.3390/nano14070637 (PMC11013762; doi:10.3390/nano14070637)
Supplement: Supplementary file 1 [file nanomaterials-14-00637-s001.zip › nanomaterials-2937445-supplementary.pdf]

nanomaterials

An Unprecedented Metal Distribution in Silica Nanoparticles Determined by Single-Particle Inductively Coupled Plasma Mass Spectrometry

Juan Han [1,2], Xu Wu [1,3], Julia Xiaojun Zhao [1,*] and David T. Pierce [1,*]

1 Department of Chemistry, University of North Dakota, 151 Cornell Street, Stop 9024, Grand Forks, ND 58202, USA; hannah.juan.han@nmt.edu (J.H.); steven.wu01@usd.edu (X.W.)
2 New Mexico Institute of Mining & Technology, 801 Leroy Place, Socorro, NM 87801, USA
3 Department of Chemistry, University of South Dakota, 414 E. Clark St., Vermillion, SD 57069, USA
* Correspondence: julia.zhao@und.edu (J.X.Z.); david.pierce@und.edu (D.T.P.); Tel.: +1-701-777-3610 (J.X.Z.); +1-701-777-2942 (D.T.P.)

S1. Abbreviations and variables used in this manuscript

materials, methods, and descriptions

NPs	nanoparticles
MNPs	metal-containing nanoparticles
AuNPs	gold nanoparticles
Ru-SiO_2 NPs	tris(bipyridyl)ruthenium(II)-doped silica nanoparticles
TEOS	tetraethyl orthosilicate
ICP-MS	inductively coupled plasma mass spectrometry/spectrometer
spICP-MS	single-particle inductively coupled plasma mass spectrometry
KED	kinetic energy discrimination mode
STDS	high-sensitivity standard acquisition mode
TEM	transmission electron microscopy/microscope
DI	deionized

constants

N_A	atoms mol^{-1}	Avogadro's number
M_x	g mol^{-1}	molar mass of species x
r_x	g cm^{-3}	density of species x

prepared, controlled, or measured quantities

C_x	mg L^{-1}	mass concentration of species x
P	NPs L^{-1}	particle concentration
u	mL min^{-1}	volume flow rate
t_d	ms	dwell/integration time
τ	s	sampling interval
A_{454}		light absorbance measured at 454 nm
d_p	nm	nanoparticle diameter
r_p	nm	nanoparticle radius
$_n$		nebulizer transport efficiency
$_i$		plasma ionization efficiency for MNP
I_x	isotope counts	conventional ICP-MS counts per dwell time for ionic species x
I_p	isotope counts	spICP-MS counts per dwell time for detected NPs

calculated quantities

$\bar{r}_p$	nm	average nanoparticle radius
$\bar{V}_p$	cm^3	average nanoparticle volume
N_p		number of Ru atoms per detected Ru-SiO_2 NP
$\bar{N}_p$		average number of Ru atoms per Ru-SiO_2 NP
N_x		number of ionic species (x) entering plasma per dwell time
$\bar{I}_x$	isotope counts	conventional ICP-MS average counts per dwell time for ionic species x
$\bar{I}_{blank}$, σ_{blank}	isotope counts	spICP-MS average and standard deviation counts per dwell time for blank sample (no NPs)
R		linear correlation coefficient
m_p	A_{454} P^{-1}	Ru-SiO_2 NP calibration slope for absorbance at 454 nm
m_{free}	A_{454} mM^{-1}	ionic $[Ru(bpy)_3]^{2+}$ calibration slope for absorbance at 454 nm

S2. Digestion and conventional ICP-MS analysis of Ru-SiO_2 NPs

Triplicate stock suspensions of Ru-SiO_2 NPs with doping levels of 0, 2, and 4 were individually digested by combining 0.5 mL of a 400.0 mg L^{-1} stock Ru-SiO_2 NPs suspension with 2.0 mL of 1 M sodium hydroxide at room temperature for 24 h. Similar methods to dissolve silica NPs using NaOH, rather than hazardous HF, have been published.[1,2] To verify complete dissolution of the Ru-SiO_2 NPs, the digestate solutions were diluted to the equivalent of 5×10^8 NP L^{-1} and measured under spICP-MS conditions (main manuscript, Table 1). Each digestate was then neutralized and diluted with 2% HNO_3 to yield a final solution containing the equivalent of 10 mg NPs L^{-1}. For accuracy comparison, the same stock suspensions of Ru-SiO_2 NPs were left intact, and duplicate samples were simply diluted with 2% HNO_3 to yield the same final solution concentration of 10 mg NPs L^{-1}. Ru concentration (given in Table S1 as ppb, mg Ru L^{-1}) was determined for each sample using the same conventional ICP-MS conditions provided in the main manuscript (Table 1).

Table S1. Ru determination by conventional ICP-MS for solutions of Ru-SiO_2 NPs with different Ru-doping levels and pretreatments.

Doping Level	Pretreatment	C_{Ru}	STDEV	Remarks
		*mg Ru L^-1^*	*mg Ru L^-1^*	
0 (control)	none (NPs intact)	not detected*		
0 (control)	digested	not detected*		
2	none (NPs intact)	0.058	0.006	no significant difference
2	digested	0.061	0.004	
4	none (NPs	0.066	0.015	no significant

[1] Leen C. J. Thomassen, L.C.J; Aerts, A.; Rabolli, V.; Lison, D.; Gonzalez, L.; Kirsch-Volders, M.; Napierska, D.; Hoet, P.H.; Kirschhock, C.E.A, Johan A. Martens, J.A. Synthesis and characterization of stable monodisperse silica nanoparticle sols for in vitro cytotoxicity testing. *Langmuir* **2010**, *26*, 328-335.

[2] Liu, Y; Wei Pan, W.; Wang, M; Zhang, K.; Zhang, H.; Huang, B.; Zhang, W.; Tan, Q.; Miao, A. Silica nanoparticle size determines the mechanisms underlying the inhibition of iron oxide nanoparticle uptake by Daphnia magna. *Environ. Sci. Technol.* **2024**, *58*, 1, 751-759

	intact)			difference
4	digested	0.066	0.006	

* Result was below limit of detection, 0.0036 mg Ru L^{-1}.

S3. Transport efficiency method and results for AuNPs of various sizes.

Transport efficiency is defined as the amount of analyte entering the plasma relative to the amount of analyte delivered to the nebulizer and spray chamber within a defined measurement period. Only a small fraction of liquid sample pumped into the ICP-MS nebulizer actually enters the plasma because all large microdroplets formed by the nebulizer collide with the walls of the spray chamber and are carried away as waste. Consistent with literature procedures [36], transport efficiency (η_n) was determined with AuNP solutions of known particle number concentration using the particle frequency method.[3] In this method, the number of AuNPs counted by spICP-MS within a certain sampling period is divided by the number of particles contained within the sampled volume of solution and multiplied by 100. The spICP-MS conditions were the same as those given in Table 1 (main manuscript), except a longer dwell time of 50 ms and lower particle number concentration of 5.00×10^6 NP $^{-1}$L was used for all transport efficiency determinations. These changes were made to minimize particle counting errors due to split- and multiple-particle measurements. No significant difference was found between the transport efficiencies determined for 30 nm, 60 nm, and 150 nm AuNPs, so an averaged value of 8.9 ± 0.1% was used for all samples. Accuracy of this value was confirmed by performing measurements of particle concentrations using 30 nm AuNP

[3] Geiss, O.; Bianchi, I.; Bucher, G.; Verleysen, E.; Brassinne, F.; Mast, J.; Loeschner, K.; Givelet, L.; Cubadda, F.; Ferraris, F.; et al. Determination of the Transport Efficiency in spICP-MS Analysis Using Conventional Sample Introduction Systems: An Interlaboratory Comparison Study. *Nanomaterials* **2022**, *12*, 725.

standards of known concentration. Two-tailed, one-sample t-test comparisons (significance level a = 0.05) between prepared particle concentration and corresponding particle concentration measurements in triplicate (e.g., 5.00 × 10^4 NPs L^{-1} vs (4.98 ± 0.16) × 10^4 NPs L^{-1}, respectively) showed no significant deviation (null confirmed, $p = 0.86 > a$).

S4. Ionization efficiency method and results for MNPs.

Particle ionization efficiency (η_i) for AuNPs with diameters of 30, 60 and 150 nm and Ru-SiO_2 NPs with doping levels 1-4 was measured for each type of NP in triplicate using ICP-MS conditions given in the main manuscript (main manuscript, Table 1).

Consistent with literature procedures [36], η_i was measured for each Au NP size with the ICP-MS instrument operating in conventional mode rather that single-particle mode. First, stock suspensions were digested by combining 0.5 mL of 50.0 mg/L AuNPs (either 30 nm, 60 nm, or 150 nm diameter) with 2.0 mL aqua regia solution at room temperature overnight. This step oxidized and dissolved the AuNPs to ionic Au. To verify complete dissolution of the AuNPs, the digestate solutions were diluted to the equivalent of 5 × 10^7 NP L^{-1} and measured under spICP-MS conditions (main manuscript, Table 1). To determine η_i for each Au NP size, the digestate solutions were diluted with 2% HNO_3 to yield a final solution containing the equivalent of 1 mg NPs L^{-1} and measured under conventional ICP-MS conditions (main manuscript, Table 1). Undigested AuNPs were also diluted to the same 1 mg NPs L^{-1} with 2% HNO_3 and measured under the same conventional ICP-MS conditions. Percent ionization efficiency (η_i) for each particle diameter was then determined by the ratio of ^{197}Au counts s^{-1} for undigested AuNPs versus digested AuNPs and multiplied by 100. Average values for the triplicate measurements are given in Table S2.

Table S2. Percent ionization efficiency (η_i) for each AuNP diameter by conventional ICP-MS using

1 mg NPs L^{-1} solutions of digested and undigested AuNPs.

**AuNP diameter**	**Average η_i (N=3)**	**STDEV**
nm	%	%
30	81	5
60	76	5
150	65	2

Only the 150 nm AuNP ionization efficiency was used in this manuscript (via Eq. 6) to generate the histogram plot of counts versus N_p (number of Au atoms per NP) shown in Fig. 8 of the main manuscript. Comparing the center of the histogram peak (600 x 10^5 Au atoms per NP) to values of *average* Au atoms per NP ($\overline{N}_p$) determined by TEM (620 x 10^5 Au atoms per NP) and conventional ICP-MS (603 x 10^5 Au atoms per NP) demonstrated no statistically significant differences and confirmed the accuracy of this ionization efficiency.

Particle ionization efficiency (η_i) for Ru-SiO_2 NPs was measured in a manner similar to AuNPs, with Ru-SiO_2 NP samples first digested to ionic Ru and ICP-MS measurement with the instrument operating in conventional mode rather that single-particle mode. Digestion of the Ru-SiO_2 NP samples and verification thereof were performed as previously described in Section 2 (Digestion and conventional ICP-MS analysis of Ru-SiO_2 NPs). To determine η_i for each Ru-SiO_2 NP doping level, the digestate solutions were neutralized and diluted with 2% HNO_3 to yield a final solution containing the equivalent of 10 mg NPs L^{-1} and measured under conventional ICP-MS conditions (main manuscript, Table1). Undigested Ru-SiO_2 NPs were also diluted to the same 10 mg NPs L^{-1} with 2% HNO_3 and measured under the same conventional ICP-MS conditions. Percent ionization efficiency (η_i) for each particle diameter was then determined by the ratio of ^{102}Ru counts s^{-1} for undigested Ru-SiO_2 NPs versus digested Ru-SiO_2 NPs and multiplied by 100. In all cases, no

significant difference was found between the ^{102}Ru counts s^{-1} of digested and undigested Ru-SiO_2 NPs (e.g., Table S1). Hence, the particle ionization efficiency for all of the Ru-SiO_2 NP doping levels was 100%.
